# [Amino­(iminio)meth­yl]phospho­nate

**DOI:** 10.1107/S1600536810032083

**Published:** 2010-08-18

**Authors:** Ting-Hai Yang, Wei Zhuang, Wei Wei, Yong-Bing Yang, Qiang Chen

**Affiliations:** a High-Tech Institute of Nangjing University, Changzhou 213164, People’s Republic of China; b School of Chemistry & Chemical Engineering, Changzhou University, Changzhou 213164, People’s Republic of China

## Abstract

The title compound, CH_5_N_2_O_3_P, exists as a zwitterion. The N atom of the imino group is protonated and the phospho­nic acid group is deprotonated. The mol­ecular geometry about the central C atom of this zwitterionic species was found to be strictly planar with the sum of the three angles about C being precisely 360°. In the crystal, the mol­ecules are inter­linked by O—H⋯O and N—H⋯O hydrogen-bonding inter­actions, forming a three-dimensional supra­molecular network structure.

## Related literature

For background to phospho­nic acid and metal phospho­nate compounds, see: Ayyappan *et al.* (2001 ▶); Clearfield (1998 ▶); Haga *et al.* (2007 ▶); Vivani *et al.* (2008 ▶); Bao *et al.* (2007 ▶); Cave *et al.* (2006 ▶); Cao *et al.* (1992 ▶); Ma *et al.* (2006 ▶, 2008 ▶). For a related structure, see Makarov *et al.* (1999 ▶).
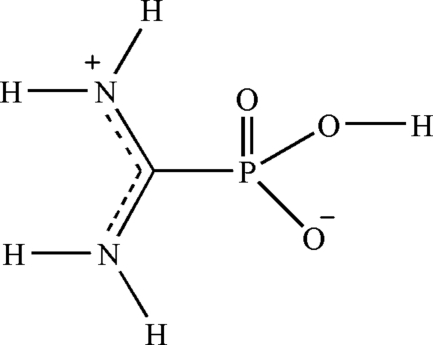


## Experimental

### 

#### Crystal data

CH_5_N_2_O_3_P
*M* *_r_* = 124.04
Triclinic, 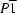

*a* = 4.8559 (17) Å
*b* = 5.910 (2) Å
*c* = 8.101 (3) Å
α = 99.570 (6)°
β = 90.784 (6)°
γ = 101.546 (6)°
*V* = 224.36 (14) Å^3^
*Z* = 2
Mo *K*α radiation
μ = 0.50 mm^−1^
*T* = 296 K
0.20 × 0.18 × 0.16 mm

#### Data collection

Bruker SMART APEX CCD diffractometer
Absorption correction: multi-scan (*SADABS*; Bruker, 2007 ▶) *T*_min_ = 0.907, *T*_max_ = 0.924
1324 measured reflections
855 independent reflections
840 reflections with *I* > 2σ(*I*)
*R*_int_ = 0.014

#### Refinement

*R*[*F* ^2^ > 2σ(*F* ^2^)] = 0.034
*wR*(*F* ^2^) = 0.161
*S* = 1.01
855 reflections
64 parameters
H-atom parameters constrained
Δρ_max_ = 0.62 e Å^−3^
Δρ_min_ = −0.77 e Å^−3^

### 

Data collection: *SMART* (Bruker, 2007 ▶); cell refinement: *SAINT* (Bruker, 2007 ▶); data reduction: *SAINT*; program(s) used to solve structure: *SHELXTL* (Sheldrick, 2008 ▶); program(s) used to refine structure: *SHELXTL*; molecular graphics: *DIAMOND* (Brandenburg, 1999 ▶); software used to prepare material for publication: *SHELXTL*.

## Supplementary Material

Crystal structure: contains datablocks I, global. DOI: 10.1107/S1600536810032083/jj2049sup1.cif

Structure factors: contains datablocks I. DOI: 10.1107/S1600536810032083/jj2049Isup2.hkl

Additional supplementary materials:  crystallographic information; 3D view; checkCIF report

## Figures and Tables

**Table 1:** Hydrogen-bond geometry (Å, °)

*D*—H⋯*A*	*D*—H	H⋯*A*	*D*⋯*A*	*D*—H⋯*A*
O3—H3*B*⋯O1^i^	0.96	1.75	2.611 (4)	147
N1—H1*A*⋯O2^ii^	0.86	2.06	2.903 (4)	167
N2—H2*A*⋯O1^ii^	0.86	2.09	2.924 (4)	164
N1—H1*B*⋯O2^iii^	0.86	2.02	2.812 (4)	153
N2—H2*B*⋯O3^iv^	0.86	2.21	3.008 (4)	154

Symmetry codes: (i) 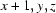; (ii) 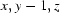; (iii) 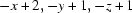; (iv) 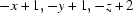.

**Fig. 1.**
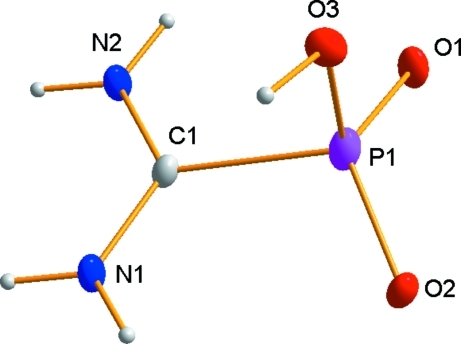
Molecular structure of the title compound, (NH2)2CPO3H, showing 50% probability displacement ellipsoids

**Fig. 2.**
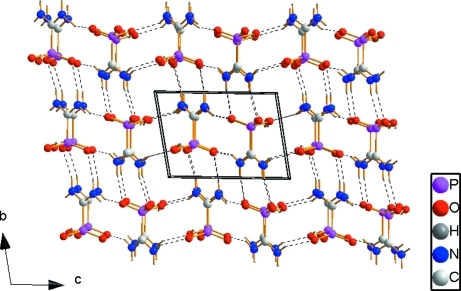
The cell packing diagram for the title compound viewed down the a axis.

## supplementary crystallographic information

### Comment

In the last decade considerable attention has been afforded to the synthesis of metal phosphonates due to their potential applications in ion-exchange and sorption, catalysis, magnetism and sensors (Ayyappan *et al.*, 2001; Clearfield, 1998; Haga *et al.*, 2007; Vivani *et al.*, 2008; Bao *et al.*, 2007; Cave *et al.*, 2006; Cao *et al.*, 1992; Ma *et al.*, 2006, 2008). In order to synthseize metal phosphonates with novel structures and properties, many kinds of phosphonic acid ligands have been used.  In order to study the crystal structure of phosphonic acid, we synthesized and determined the structure of the title compound (Fig. 1).  As shown in Scheme 1, the molecular exists as a zwitterion, the imino group being protonated and the phosphonic acid group being deprotonated. The molecular geometry about the central C atom is strictly planar with the sum of the three angles about C being precisely 360°. The three bonds about the central carbon atom consist of two nearly equivalent C–N1 and C–N2 distances of 1.299 (5) Å and 1.314 (5) Å, respectively, and a C–P bond distance of 1.845 (3) Å. These two C–N bonds are considerably shorter than a typical C–N single bond distance of 1.47 Å,  Similar zwitterions have been formed by other aminoiminomethanesulfonic acids (Makarov *et al.*,1999). The P–O distances in these compounds range from 1.4872 (2) Å to 1.5872 (2) Å. By comparision of individual P—O distances, the H atom can be located on O3. In our crystal structure, three intermolecular hydrogen-bond interactions exist, *viz*. between the N atom and the phosphonate O atom [N1—H1A···O2, N2—H2A···O1, N1—H1B···O2, N2—H2B···O3], and between two phosphonate O atoms [O3—H3B···O1] (Table 1).  Thus the molecules are interlinked by these intermolecular hydrogen bonds, forming a three-dimensional supramolecular network structure (Fig.2).

### Experimental

All solvents and chemicals were of analytical grade and were used without further purification. The title compound was prepared by the following reaction: A sample of 2,4,6-tri-(phosphonate ethyl)-1,3,5-triazine (9.8 g, 20 mmol) was dissolved in 6 mol/ ml HCl (20 ml), The mixture was heated (100 °C, 10 h) and then evaporated to dryness leaving a white solid. Crystallization was carried out by dissolution of 0.62 g of the title compound (about 0.5 mmol) in 10 ml water, followed by evaporation at room temperature. After two weeks, colorless block crystals obtained.

### Refinement

All non-hydrogen atoms were refined anisotropically, whereas the positions of all H atoms bonded to nitrogen were fixed geometrically (N—H = 0.86 Å), and included in the refinement in the riding mode, with Uĩso~(H) = 1.2U~eq~(N). The H atom in P—O—H was located in a difference Fourier map and refined with a distance restraint of O—H = 0.96 Å, and with Uĩso~(H) = 1.5U~eq~(O).

### Crystal data

**Table:**

CH_5_N_2_O_3_P	*Z* = 2
*M**_r_* = 124.04	*F*(000) = 128
Triclinic, *P*1	*D*_x_ = 1.836 Mg m^−^^3^
Hall symbol: -P 1	Mo *K*α radiation, λ = 0.71073 Å
*a* = 4.8559 (17) Å	Cell parameters from 1436 reflections
*b* = 5.910 (2) Å	θ = 2.6–30.3°
*c* = 8.101 (3) Å	µ = 0.50 mm^−^^1^
α = 99.570 (6)°	*T* = 296 K
β = 90.784 (6)°	Block, colorless
γ = 101.546 (6)°	0.20 × 0.18 × 0.16 mm
*V* = 224.36 (14) Å^3^	

### Data collection

**Table:**

Bruker SMART APEX CCD diffractometer	855 independent reflections
Radiation source: fine-focus sealed tube	840 reflections with *I* > 2σ(*I*)
graphite	*R*_int_ = 0.014
phi and ω scans	θ_max_ = 26.0°, θ_min_ = 2.6°
Absorption correction: multi-scan (*SADABS*; Bruker, 2007)	*h* = −5→5
*T*_min_ = 0.907, *T*_max_ = 0.924	*k* = −6→7
1324 measured reflections	*l* = −9→9

### Refinement

**Table:**

Refinement on *F*^2^	Primary atom site location: structure-invariant direct methods
Least-squares matrix: full	Secondary atom site location: difference Fourier map
*R*[*F*^2^ > 2σ(*F*^2^)] = 0.034	Hydrogen site location: inferred from neighbouring sites
*wR*(*F*^2^) = 0.161	H-atom parameters constrained
*S* = 1.01	*w* = 1/[σ^2^(*F*_o_^2^) + (0.1046*P*)^2^ + 0.7669*P*] where *P* = (*F*_o_^2^ + 2*F*_c_^2^)/3
855 reflections	(Δ/σ)_max_ < 0.001
64 parameters	Δρ_max_ = 0.62 e Å^−^^3^
0 restraints	Δρ_min_ = −0.77 e Å^−^^3^

### Special details

**Table:**

Geometry. All e.s.d.'s (except the e.s.d. in the dihedral angle between two l.s. planes) are estimated using the full covariance matrix. The cell e.s.d.'s are taken into account individually in the estimation of e.s.d.'s in distances, angles and torsion angles; correlations between e.s.d.'s in cell parameters are only used when they are defined by crystal symmetry. An approximate (isotropic) treatment of cell e.s.d.'s is used for estimating e.s.d.'s involving l.s. planes.
Refinement. Refinement of *F*^2^ against ALL reflections. The weighted *R*-factor *wR* and goodness of fit *S* are based on *F*^2^, conventional *R*-factors *R* are based on *F*, with *F* set to zero for negative *F*^2^. The threshold expression of *F*^2^ > σ(*F*^2^) is used only for calculating *R*-factors(gt) *etc*. and is not relevant to the choice of reflections for refinement. *R*-factors based on *F*^2^ are statistically about twice as large as those based on *F*, and *R*- factors based on ALL data will be even larger.

### Fractional atomic coordinates and isotropic or equivalent isotropic displacement parameters (Å^2^)

**Table:**

	*x*	*y*	*z*	*U*_iso_*/*U*_eq_	
P1	0.66113 (16)	0.60637 (13)	0.74674 (10)	0.0149 (4)	
O1	0.3963 (5)	0.6733 (4)	0.8092 (3)	0.0225 (6)	
O2	0.7837 (5)	0.6917 (4)	0.5956 (3)	0.0224 (6)	
O3	0.8828 (5)	0.6695 (5)	0.9016 (3)	0.0224 (6)	
H3B	1.0481	0.6105	0.8696	0.034*	
N1	0.7663 (7)	0.1822 (5)	0.6080 (4)	0.0224 (7)	
H1A	0.7485	0.0324	0.5929	0.027*	
H1B	0.8918	0.2651	0.5566	0.027*	
N2	0.4088 (7)	0.1649 (5)	0.7899 (4)	0.0229 (7)	
H2A	0.3854	0.0148	0.7776	0.027*	
H2B	0.3047	0.2374	0.8554	0.027*	
C1	0.6036 (7)	0.2834 (6)	0.7084 (4)	0.0168 (7)	

### Atomic displacement parameters (Å^2^)

**Table:**

	*U*^11^	*U*^22^	*U*^33^	*U*^12^	*U*^13^	*U*^23^
P1	0.0130 (6)	0.0125 (5)	0.0216 (6)	0.0045 (3)	0.0061 (4)	0.0070 (3)
O1	0.0156 (13)	0.0197 (13)	0.0350 (14)	0.0076 (10)	0.0086 (10)	0.0073 (10)
O2	0.0245 (13)	0.0199 (13)	0.0271 (14)	0.0067 (10)	0.0113 (10)	0.0124 (10)
O3	0.0150 (13)	0.0271 (14)	0.0247 (13)	0.0047 (10)	0.0029 (10)	0.0032 (10)
N1	0.0243 (16)	0.0139 (14)	0.0318 (17)	0.0057 (12)	0.0124 (13)	0.0088 (12)
N2	0.0263 (16)	0.0145 (14)	0.0299 (17)	0.0052 (12)	0.0138 (13)	0.0074 (12)
C1	0.0170 (16)	0.0147 (15)	0.0207 (16)	0.0044 (12)	0.0026 (12)	0.0073 (12)

### Geometric parameters (Å, °)

**Table:**

P1—O2	1.487 (2)	N1—H1A	0.8600
P1—O1	1.490 (3)	N1—H1B	0.8600
P1—O3	1.587 (3)	N2—C1	1.314 (5)
P1—C1	1.845 (3)	N2—H2A	0.8600
O3—H3B	0.9600	N2—H2B	0.8600
N1—C1	1.299 (5)		
			
O2—P1—O1	119.46 (15)	C1—N1—H1B	120.0
O2—P1—O3	111.94 (15)	H1A—N1—H1B	120.0
O1—P1—O3	106.97 (15)	C1—N2—H2A	120.0
O2—P1—C1	108.20 (15)	C1—N2—H2B	120.0
O1—P1—C1	107.89 (15)	H2A—N2—H2B	120.0
O3—P1—C1	100.70 (15)	N1—C1—N2	122.4 (3)
P1—O3—H3B	109.3	N1—C1—P1	118.7 (3)
C1—N1—H1A	120.0	N2—C1—P1	118.8 (3)
			
O2—P1—C1—N1	28.6 (3)	O2—P1—C1—N2	−154.6 (3)
O1—P1—C1—N1	159.2 (3)	O1—P1—C1—N2	−24.1 (3)
O3—P1—C1—N1	−88.9 (3)	O3—P1—C1—N2	87.8 (3)

### Hydrogen-bond geometry (Å, °)

**Table:**

*D*—H···*A*	*D*—H	H···*A*	*D*···*A*	*D*—H···*A*
O3—H3B···O1^i^	0.96	1.75	2.611 (4)	147.
N1—H1A···O2^ii^	0.86	2.06	2.903 (4)	167.
N2—H2A···O1^ii^	0.86	2.09	2.924 (4)	164.
N1—H1B···O2^iii^	0.86	2.02	2.812 (4)	153.
N2—H2B···O3^iv^	0.86	2.21	3.008 (4)	154.

Symmetry codes: (i) *x*+1, *y*, *z*; (ii) *x*, *y*−1, *z*; (iii) −*x*+2, −*y*+1, −*z*+1; (iv) −*x*+1, −*y*+1, −*z*+2.
